# Evaluation of Combining Transrectal Biplane Ultrasonography with Sound Touch Elastography in Preoperative T-Staging of Rectal Cancer

**DOI:** 10.2174/0115734056366881250625114331

**Published:** 2025-06-30

**Authors:** Yan Zhang, Lu Liang, Huachong Ma, Jiagang Han, Xiuzhang Lyu, Huiyu Ge

**Affiliations:** 1 Department of Ultrasound Medicine, Beijing Chaoyang Hospital, Capital Medical University, Beijing, 100020, China; 2 Department of Radiology, Beijing Chaoyang Hospital, Capital Medical University, Beijing, 100020, China; 3 Department of General Surgery, Beijing Chaoyang Hospital, Capital Medical University, Beijing, 100020, China

**Keywords:** Transrectal biplane ultrasonography, Sound touch elastography, Rectal cancer, Staging diagnosis, Stiffness ratio, Rectal cancer, Computer tomography, Magnetic resonance imaging

## Abstract

**Introduction:**

An accurate staging diagnosis of rectal cancer holds crucial importance in determining the appropriate treatment plan for patients.

**Aim:**

To evaluate the application of transrectal biplane ultrasonography combined with Sound Touch Elastography (STE) technology in preoperative uT stage of rectal cancer.

**Methods:**

A retrospective analysis was conducted on the ultrasonographic data of 32 patients. The STE values within the tumor and the adjacent peritumoral fat tissue were recorded, and the ratio of STE values between adjacent and distant peritumoral fat tissues was defined as the Stiffness Ratio (SR).

**Results:**

The STE values were not statistically significantly different between the high and low pT stage groups within tumors (P > 0.05). However, there were statistically significant differences in the STE values of the adjacent peritumoral fat tissue and the SR between the two groups (*P* < 0.05). Binary logistic regression analysis showed that the SR was a relevant factor in distinguishing high and low pT stages of rectal cancer. The optimal cut-off value of the SR was 1.915, with a sensitivity of 95.7% and a specificity of 88.9% in predicting high pT stages of rectal cancer. The consistency observed between traditional TRUS and pathological staging in differentiating between high and low pT stages of rectal cancer was moderate. However, the incorporation of SR had enhanced this consistency to a favorable level.

**Conclusion:**

The combination of TRUS and STE technology enhanced the accuracy of pT stage in rectal cancer, with SR serving as a critical indicator for predicting high pT stages and constituting a valuable supplement to traditional TRUS.

## 1 INTRODUCTION

Rectal cancer is one of the common malignancies in the digestive system. According to data published by the American Cancer Society in 2024 [1], rectal cancer ranks third among newly diagnosed malignancies in the digestive system. Since a significant number of rectal cancer deaths were previously misclassified as colon cancer, the society combined the mortality rates of colon and rectal cancers for statistical purposes. The combined mortality rate now ranks second among all malignancies. In the diagnosis and treatment of rectal cancer, the selection of treatment options heavily relies on the tumor stage. Inadequate staging or over-staging can significantly impact patient survival and prognosis. According to the National Comprehensive Cancer Network (NCCN) Clinical Practice Guidelines in Oncology for Rectal Cancer, patients with T1−T2 stage rectal cancer may consider local excision surgery, while those with T3−T4 stage are recommended to receive preoperative neoadjuvant chemoradiotherapy to improve the success rate of surgical resection and reduce the postoperative local recurrence rate [2]. Therefore, an accurate assessment of the T3 stage is crucial for determining appropriate treatment strategies.

Currently, the staging diagnosis of rectal cancer relies primarily on various technologies such as Computer Tomography (CT), Magnetic Resonance Imaging (MRI), and Transrectal Ultrasound (TRUS). CT has a certain value in assessing tumor size, location, and surrounding conditions. However, it has limitations in displaying the fine layers of the rectal wall and is used mainly to evaluate adjacent tissue invasion and distant metastasis. MRI, with its excellent soft tissue and spatial resolution, clearly delineates the various layers of the rectal wall. It has become the preferred imaging technique for staging locally advanced rectal cancer and restaging after neoadjuvant therapy, and is especially suitable for the diagnosis of T3 stages and higher. However, MRI is less sensitive than TRUS [3] in the early diagnosis of T1 and T2 tumors and has drawbacks, such as high cost, loud noise, and long examination time. It is contraindicated for specific patient populations, such as those with internal metallic implants or claustrophobia. TRUS has been used in the diagnosis and treatment of rectal cancer for over 30 years. It has become an important tool for assessing tumor invasion depth and adjacent organ involvement because it offers the advantages of convenience, high resolution, and the fact that it does not require radiation. In particular, ultrasound elastography provides a new perspective for differentiating benign and malignant tumors by reflecting the mechanical properties of biological tissues. Among this technology, Shear Wave Elastography (SWE) enhances assessment accuracy and repeatability by quantifying tissue stiffness through the measurement of shear wave velocity and calculating Young’s modulus, independently of operator-applied pressure.

In recent years, domestic and international research has predominantly used intracavitary end-fire convex array probes combined with SWE technology for rectal cancer staging diagnosis [4-6]. However, some studies have also explored the use of Strain Elastography (SE) with 360° intracavitary probes or intracavitary biplane probes in linear array mode [7, 8]. Traditional SE technology relies on manual compression or axial displacement of tissues caused by physiological movements (such as heartbeat, respiration, *etc*.), but can only reflect tissue strain information through deformation, without providing Young’s modulus values. In contrast, SWE is more advanced, as it emits acoustic radiation force pulses to stimulate the tissue to generate transverse shear waves and uses color-coded technology to reflect the elasticity map of the lesion tissue in real time. It can measure shear wave velocity to calculate Young's modulus and assess tissue stiffness. Sound Touch Elastography (STE) is an ultrasound imaging technology developed from the principles of two-dimensional SWE [9]. It uses ultra-wideband beam tracking technology and rapid single-shot high-frequency signal processing to display real-time two-dimensional color-coded images of tissue stiffness within the region of interest, thereby accurately reflecting the elastic modulus of tissues over a larger area and in a shorter time [10, 11]. Notably, there has been a notable lack of research reports on the application of an intracavitary biplane probe in linear array mode, in combination with STE technology, for staging diagnosis in rectal cancer. Consequently, the aim of this study was to assess the accuracy of preoperative T staging diagnosis of rectal cancer by utilizing transrectal biplane ultrasound in conjunction with STE technology, and to thoroughly examine the factors that influenced the staging diagnosis of rectal cancer.

## 2 METHODS

### 2.1 Study Subjects

**Fig. (1):**
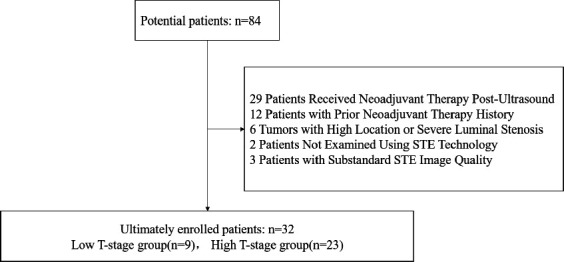
Flow chart of the inclusion of patients.

This study included the case records of 84 patients with rectal cancer who underwent staging diagnosis in the Ultrasound Medical Department from August 2022 to January 2024. The specific patient inclusion criteria were as follows: histopathologically confirmed rectal cancer and having undergone preoperative transrectal biplane ultrasound for staging diagnosis. The patient exclusion criteria included: having received neoadjuvant therapy after ultrasound examination, a history of prior neoadjuvant therapy, having tumors located higher up or accompanied by severe luminal stenosis, making it impossible to assess the tumor comprehensively using transrectal biplane ultrasound, and not being examined using STE technology or the image quality obtained by STE technology not meeting diagnostic requirements. Ultimately, 32 patients were included. According to the surgical pathological stage, patients with pT1 and pT2 stages were classified into the low pT stage group, while those with pT3 and pT4 stages were classified into the high pT stage group. This study involved prospective data recording and retrospective analysis. It strictly adhered to medical ethical principles and obtained approval from the medical ethics committee, as well as written informed consent from the patients (Fig. **1**).

In this study, the Mindray ResonaR9 Color Doppler Ultrasound Diagnostic System (Shenzhen Mindray Bio-Medical Electronics Co., Ltd., Shenzhen, Guangdong Province, China) was used, equipped with the ELC13-4U intracavitary biplane probe (convex array probe with a frequency range of 3.5–9.5 MHz, and linear array probe with a frequency range of 3.2–12.8 MHz). The TRUS and STE examinations were performed using a sonographer with over 20 years of experience in gastrointestinal ultrasound diagnosis. To ensure the objectivity and accuracy of the study, the sonographer was blinded to the patients’ pathological diagnoses, other imaging results (such as MRI, CT), and colonoscopy findings during the examination.

### 2.2 TRUS Image Acquisition Method

#### 2.2.1 Pre-examination Preparation

Patients were instructed to undergo bowel cleansing 1 hour before the TRUS examination by administering 110 milliliters of glycerin enema *via* the anus. During the examination, patients were positioned in the left lateral decubitus position with their hips and knees flexed. Their right hand was used to gently separate the buttocks to fully expose the anal area. An appropriate amount of coupling agent was applied to the surface of the probe, and subsequently, the probe was covered with a latex sheath. The air inside the latex sheath was carefully removed to avoid interference, and an additional layer of coupling agent was applied outside the sheath to ensure optimal contact and imaging quality.

#### 2.2.2 Transrectal Biplane Ultrasound Linear Array Mode

The sonographer delicately inserted the intracavitary biplane probe into the rectal cavity. Subsequently, the probe was switched to the high-frequency linear array mode to exhibit the maximal cross-sectional view of the tumor infiltrating the intestinal wall. Following this, the STE imaging mode was activated. The Region of Interest (ROI) was configured to a size of 4×3 cm, encompassing both the tumor itself and its perimeter. The tissue stiffness was visually depicted using a color-coded scheme, with blue representing low stiffness and red indicating high stiffness. Images were captured without exerting pressure, and three separate dynamic images were saved when the Motion Stability Index (M-STB) achieved four green stars or higher, ensuring optimal stability.

### 2.3 Data Acquisition

The STE static images marked with an M-STB of five green stars were selected from the hard drive of the ultrasound system for quantitative measurements using the instrument’s built-in Q-box tool. The measurements included the STE value within the tumor, the STE value of adjacent peritumoral fatty tissue, and the Stiffness Ratio (SR) between adjacent and distant peritumoral fatty tissues.

#### 2.3.1 Measurement of STE Value within Rectal Tumors

**Fig. (2):**
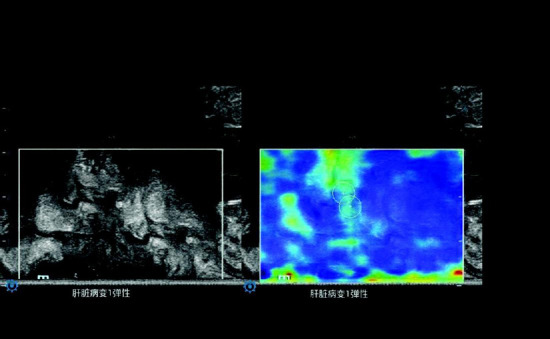
Measurement of the STE value within rectal tumor.

Dynamic images of the rectal tumor were reviewed, and the Q-box size was set to a 0.2–0.5 cm diameter circle based on the hardest area of the tumor. After two measurements were taken in each sample, the data were stored by pressing the “Print” button. A total of six measurements were conducted from three different images, and the mean STE value of the hardest part of the tumor was recorded (Fig. **2**).

#### 2.3.2 Measurement of the STE Value of Adjacent Peritumoral Fatty Tissue

**Fig. (3):**
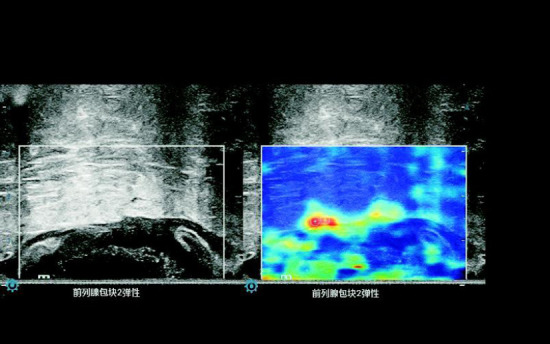
Measurement of the STE value in adjacent peritumoral fatty tissue.

Dynamic images containing the tumor edge and peritumoral fatty tissue were reviewed. A 0.2 cm diameter circular Q-box was placed on the hardest part of the peritumoral fatty tissue within 0.5 cm of the deepest tumor infiltration (typically representing a hyperechoic area) while avoiding visible blood vessels and lymph nodes. After two measurements were taken in each sample, the data were stored. Six measurements were taken from three images, and the mean STE value of the adjacent peritumoral fatty tissue was recorded (Fig. **3**).

#### 2.3.3 Calculation of STE Stiffness Ratio between Adjacent and Distant Peritumoral Fatty Tissues

**Fig. (4):**
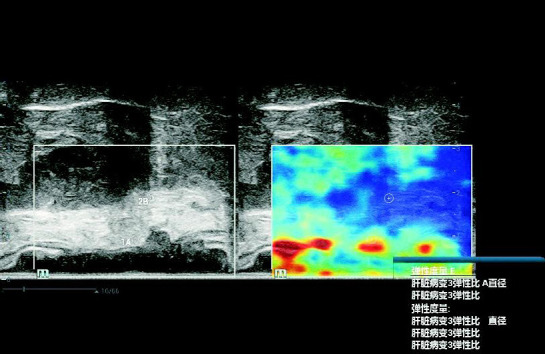
Calculation of the STE stiffness ratio between adjacent and distant peritumoral fatty tissues.

A 0.2 cm diameter circular Q-box was placed on the hardest part of the peritumoral fatty tissue within 0.5 cm of the deepest tumor infiltration (denoted as A), and another 0.2 cm diameter circular Q-box was placed on fatty tissue (denoted as B) located more than 0.5 cm deeper than A (Fig. **4**). The SR between A and B was calculated, and the average SR was recorded after three measurements. Throughout the measurement process, it was ensured that the Standard Deviation (SD) of each measurement was controlled within 20%, and the ratio of the Interquartile Range (IQR) to the Median (M) was ≤30%.

Based on the Hildebrandt staging criteria [12], two sonographers independently assessed the uT stage of rectal tumor infiltration depth. In cases where the staging results were inconsistent, a third sonographer was invited to participate in the evaluation, and the final uT stage of the rectal tumor was determined after collective discussion and consensus. Specifically, uT1 indicates tumor invasion of the mucosa or submucosa, uT2 indicates tumor invasion of the muscularis propria, uT3 denotes tumor infiltration of all layers of the intestinal wall and the surrounding perirectal fatty tissue, and uT4 indicates tumor invasion of adjacent organs or the lateral pelvic wall tissue.

### 2.4 Statistical Analysis

The collected data were analyzed using SPSS version 27.0 software. Continuous variables were expressed as medians with Interquartile Ranges (IQRs), while categorical variables were presented as frequencies (n) and percentages (%). With surgical pathological stage as the reference standard, the Mann-Whitney U test was used to assess differences between the two groups of continuous variables, and Fisher’s exact test was used to compare differences between the two groups of categorical variables. Statistical significance was considered when the two-tailed p-value was less than 0.05. Based on the statistical analysis results, parameters with statistically significant differences were selected and used to construct a logistic regression model. Receiver Operating Characteristic (ROC) curves were plotted for statistically significant parameters, and the Area Under the Curve (AUC) was calculated. Through ROC curve analysis, parameters with higher sensitivity and specificity were selected as the optimal cut-off values for distinguishing between high and low pT stages. In addition, the Kappa test was used to assess the agreement with pathological results. A Kappa value of ≥0.75 was considered to indicate good agreement, a Kappa value of 0.4–0.75, moderate agreement, and a Kappa value of ≤0.4, poor agreement.

## 3 RESULTS

### 3.1 Comparative Analysis of Baseline Characteristics between Two Patient Groups

This study ultimately included data from 32 patients, with 21 males and 11 females. The median age of the patients was 65 years, ranging from 36 to 79 years. Based on pathological T stage (pT stage), there were two patients (6.3%) in the pT1 stage, seven patients (21.9%) in the pT2 stage, 20 patients (62.5%) in the pT3 stage, and three patients (9.4%) in the pT4 stage. Patients in the pT1 and pT2 stages were classified into the low pT stage group, while those in the pT3 and pT4 stages were classified into the high pT stage group. All patients underwent postoperative pathological tissue verification, revealing that one patient had rectal neuroendocrine carcinoma, and the remaining 31 had rectal adenocarcinoma. When comparing the age, sex, and basic information on whether the anus was preserved between the two patient groups, this study found no statistically significant difference (*P* > 0.05). The clinical characteristics of the patients are presented in Table **1**.

### 3.2 Comparison of STE Values between Low and High pT Stage Groups

The STE values within the tumor did not differ significantly between the high and low pT stage groups (*P* > 0.05). However, statistically significant differences were observed in the STE values of the adjacent peritumoral adipose tissue, as well as in the SR, between the two groups (*P* < 0.05). For detailed information (Table **2**).

### 3.3 Analysis of Factors Influencing High *vs.* Low pT Stages in Rectal Cancer

Binary logistic regression analysis was carried out to assess the impact of STE values in adjacent peritumoral adipose tissue and the SR on the high *vs.* low pT stage classification of rectal cancer. In this analysis, the dependent variable was the pT stage of rectal cancer (low = 1, high = 2), while the independent variables were the STE values in adjacent peritumoral adipose tissue and the SR. The results indicated that SR was a significant factor in distinguishing between high and low pT stages of rectal cancer (*OR* = 376.27, *P* = 0.041) (*P* < 0.05) (Table **3**).

### 3.4 SR Cut-Off Value for Diagnosing High pT Stage in Rectal Cancer

**Fig. (5):**
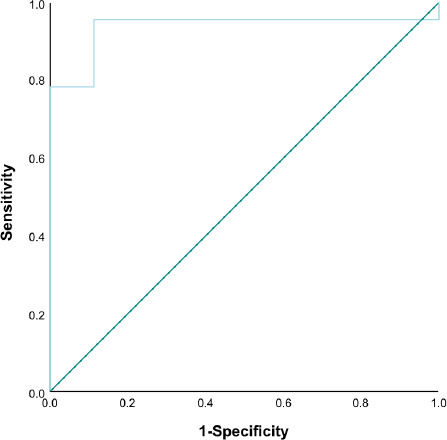
ROC Curve for predicting high pT stage in rectal cancer using SR.

The ROC curve demonstrated that the AUC for distinguishing high *vs.* low pT stages in rectal cancer using SR was 0.937. The optimal cut-off value for SR was 1.915, with a sensitivity of 95.7% and a specificity of 88.9% for predicting high pT stages in rectal cancer (Fig. **5**).

### 3.5 Application Value of TRUS vs TRUS Combined with SR in High and Low uT Stage

Using pT stages as the reference standard and traditional TRUS as the basis, for patients with a low uT stage, if the SR value was >1.915, the uT stage was upgraded to high, while if the SR value was <1.915, the stage remained unchanged. For patients with a high uT stage, if the SR value was >1.915, the uT stage remained unchanged, while if the SR value was <1.915, the stage was downgraded to low. In this study, four patients were overstaged as high uT stage *via* traditional TRUS. In addition, one patient was overstaged and one was under-staged as high uT stage *via* traditional TRUS combined with SR (Table **4**).

When comparing the consistency between traditional TRUS and pathological stage in distinguishing high and low pT stages of rectal cancer, the results showed moderate agreement between the two (Kappa = 0.642, *P* < 0.001). However, when traditional TRUS was combined with SR for the assessment of high and low pT stages of rectal cancer, a good level of agreement was demonstrated with pathological stage (Kappa = 0.845, *P* < 0.001).

## 4 DISCUSSION

Rectal cancer, the third most prevalent malignancy in the digestive system, poses a grave threat to human life and health. Precise tumor stage is essential for providing patients with the most appropriate treatment plan. TRUS—characterized by its nonradiative nature, ease of operation, cost-effectiveness, and high-resolution imaging—can distinctly delineate the layered structure of the rectal wall. As an advanced development of SWE technology, STE offers a more accurate assessment of tissue elasticity values. Previous studies had reported that STE technology was predominantly utilized for measuring the elastic modulus of tissues and organs, including the liver, spleen, kidneys, and tendons [13-16], while there was scant investigation exploration by utilizing an intracavity biplane probe in combination with STE technology in a linear array mode to quantify the Young’s modulus with rectal tumors and their adjacent adipose tissue. This represented a groundbreaking endeavor to integrate an intracavitary biplane probe with STE technology in the linear array mode.

This study indicated that, in the intracavitary biplane ultrasound linear array mode, measuring the E_max_ in the hardest region of rectal cancer did not distinctly differentiate between high and low pT stages (P > 0.05). However, in the assessment of T stage in rectal cancer, the literature exhibits varied opinions concerning the utility of tumor stiffness measurement. Chen *et al*. [17] utilized an intracavitary end-fire convex array probe integrated with SWE technology to quantify the stiffness of rectal tumors, peritumoral fat, and the SR of tumors relative to both normal rectal wall and distant perirectal fat. Their findings revealed a significant elevation in tumor stiffness, peritumoral fat stiffness, and SR of tumors compared to both normal rectal wall and distant perirectal fat, progressing from rectal adenoma to T3 rectal cancer. Similarly, Dong *et al*. [4] applied an intracavitary end-fire convex array probe paired with SWE to measure the stiffness of rectal tumors, peritumoral fat, and distant perirectal fat, observing a progressive increase in the maximum Young’s modulus (E_max)_ of tumors across T1 to T3 stages. Notably, the diagnostic accuracy of TRUS combined with tumor-fat SWE E_max_ stage surpassed that of traditional TRUS. Furthermore, Fan *et al*. [6] conducted a study using an end-fire convex array probe integrated with SWE, demonstrating that both the average and maximum stiffness values of tumors increased with advancing T stages. Conversely, Feng *et al*. [7] reported that when using an intracavitary biplane probe in linear array mode coupled with SE technology to measure the SR within tumors and surrounding fat, the diagnostic accuracy of TRUS combined with SE technology in T stage of rectal cancer did not align with excellent outcomes, particularly for early rectal cancer patients.

The discrepancies in stiffness measurements could be attributed to several factors. First, variations in Q-box selection—where some scholars manually delineate the tumor boundary while others opt for a circular area for stiffness measurement—lead to differing stiffness values due to different sampling areas. Second, SWE technology evaluation parameters lack uniformity, with some studies using the E_max_ and others using the E_mean_. Third, there are differences in probe choice and application technologies. Some studies use intracavitary end-fire convex array probes, while others use intracavitary biplane probes. Similarly, some studies incorporate SWE technology, while others use SE technology. In addition, operator experience can impact the accuracy of lesion interpretation, thereby influencing the diagnostic accuracy of elastography in the tumor T stage.

This study also demonstrated that the E_max_ of adjacent peritumoral fat tissue and the SR between adjacent and distant peritumoral fat tissues, measured by STE, were statistically significant in differentiating high and low pT stages of rectal cancer (P < 0.05). Logistic regression analysis revealed that the SR between adjacent and distant peritumoral fat tissues served as a crucial indicator for distinguishing high and low pT stage rectal cancer. Previous studies primarily limited statistical analysis to intergroup comparisons without conducting multivariate analysis, which may have contributed to differing results. This study also showed that the AUC for distinguishing high and low pT stages of rectal cancer based on SR values was 0.937, with an optimal cut-off value of 1.915. When reassessing tumor uT stage *via* traditional TRUS combined with SR values—with pathological results serving as the gold standard—it was found that one patient was overestimated into the high pT stage group and another was underestimated into the low pT stage group. In contrast, when only traditional TRUS was used, four patients were overestimated into the high pT stage group. Notably, for patients with low pT stage tumors, traditional TRUS frequently misclassifies them as high pT stage due to adjacent inflammation, locally retained secretions, or connective tissue hyperplasia reactions. These reactive changes manifest as hypoechoic features on traditional TRUS, and the outer layer of the rectal wall exhibits an irregular morphology resembling that of a transmural tumor [17]. Therefore, in clinical practice, these factors should be considered thoroughly in the assessment results of TRUS. The SR value can be used to form a more comprehensive judgment, enhancing the accuracy of the high and low pT stage tumor assessments.

Based on the comparison results, this study revealed a certain degree of inconsistency between traditional TRUS and pathological stage in determining the high and low pT stages of rectal cancer. The consistency between traditional TRUS and pathological stage was moderate (Kappa = 0.642, P < 0.001). However, a good level of consistency was reached between traditional TRUS combined with SR and pathological stage (Kappa = 0.845, P < 0.001). This suggested that combining the SR value could more accurately distinguish between high and low pT stages of rectal cancer in the lateral segment. Notably, the SR parameter, which had not been addressed in previous reports, was closely related to the infiltration status of tumor tissue. Several weeks after tumor infiltration occurs in fat, cancer cells enter adipocytes and stimulate the proliferation of interstitial fibroblasts and collagen fibers due to the aggressiveness and infiltrative nature of tumor cells. These structural changes in tumor tissue are reflected in the changes in tissue stiffness. Therefore, by measuring the ratio of stiffness between the peritumoral fat tissue and remote fat tissue, the presence or absence of tumor tissue infiltration could be assessed objectively.

This study had several limitations. Firstly, due to the concealed manifestations of early rectal cancer, the majority of patients were diagnosed at a locally advanced stage, consequently constraining the sample size that could be obtained for this research. Additionally, some patients who were classified with a high uT stage might have undergone neoadjuvant therapy, whereas this study employed pathological examination of resected specimens as the definitive criterion, which similarly impeded the augmentation of the sample size. Secondly, ethical considerations precluded the imposition of unnecessary examination discomfort through repeated operations by multiple practitioners on the same patient. Consequently, comparisons between different physicians were not conducted, potentially impacting the stability and reproducibility of the results. Thirdly, the intracavitary biplane probe had a limited depth of exploration, reaching only lesions within 14 cm above the anal verge, thus preventing the assessment of segments involving the sigmoid colon. Finally, this study only measured the STE values at the hardest points of the tumor and peritumoral fat tissue, as well as the STE values of the remote peritumoral fat tissue. Future research endeavors could delve deeper into key parameters, such as the minimum STE, average STE, and shear wave velocity values of the tumor and peritumoral fat tissue. Given the limited sample size of this study, we anticipate that larger-scale research involving more diverse patient populations will be conducted in the future to further validate and consolidate the findings.

## CONCLUSION

In summary, the integrated use of transrectal biplane ultrasound and STE technology enhanced the accuracy of the uT stage in rectal cancer. Notably, SR emerged as a pivotal indicator for predicting high pT stages, playing a crucial role in improving the accuracy of rectal cancer uT stage. Consequently, this technology served as a valuable adjunct to traditional transrectal ultrasound, aiding clinicians in developing treatment plans that are more personalized and aligned with the circumstances of the individual patient.

## Figures and Tables

**Table 1:** Comparison of baseline characteristics between the two patient groups.

**Indicators**	**Low pT stage Group(N=9)**	**High pT stage Group(N=23)**	**Z-value**	***P*-value**
Age	65(60,70)	70(58,73)	-0.819	0.413
Sex				1.000
Male	6(66.7%)	15(65.2%)		
Female	3(33.3%)	8(34.8%)		
Anus-preserving				0.648
Yes	8(88.9%)	18(78.3%)		
No	1(11.1%)	5(21.7%)		

**Note:** pT: Pathological T stage. Data were represented as median (interquartile range).

**Table 2:** Comparison of ultrasonic characteristics between the two patient groups.

**Characteristics**	**Low pT stage Group**	**High pT stage Group**	***Z/F* value**	***P*-value**
STE Value within Rectal Tumor(kPa)	39.03(26.99,43.97)	43.36(26.99,50.27)	-0.775	0.438
STE Value in Adjacent Peritumoral Fatty Tissue(kPa)	10.98(9.78,20.03)	33.01(23.67,46.08)	-3.458	<0.001*
SR	1.08(1.01,1.43)	3.15(2.44,3.15)	-3.793	<0.001*

**Note:** *The difference was statistically significant (*P* < 0.05). STE: Sound touch elastography. SR: The stiffness ratio of STE values between adjacent and distant peritumoral fat tissues. Data were represented as median (interquartile range).

**Table 3:** Analysis of factors influencing the high and low pT stage classification in rectal cancer.

**Factors**	**B value**	**SE**	**Wals**	***p*-value**	***OR* value**	**95% Confidence Interval (CI) for the OR Value**
STE Value in Adjacent Peritumoral Fatty Tissue(kPa)	-0.214	0.180	1.422	0.233	0.807	(0.568,1.148)
SR	5.930	2.896	4.195	0.041*	376.270	(1.291,109698.040)

**Note:** *The difference was statistically significant (*P* < 0.05). SE: Standard error of the B value. STE: Sound touch elastography. SR: The stiffness ratio of STE values between adjacent and distant peritumoral fat tissues.

**Table 4:** Comparison of TRUS stage, TRUS combined with SR stage, and pT stage.

**Indicators**	**Low pT Stage Group**	**High pT Stage Group**
uT Stage		
Low uT Stage Group	5(100%)	0(0%)
High uT Stage Group	4(14.8%)	23(85.2%)
TURS+SR Stage		
Low uT Stage Group	8(88.9%)	1(11.1%)
High uT Stage Group	1(4.3%)	22(95.7%)

**Abbreviations:** uT stage: Ultrasound T stage. pT: Pathological T stage.

## Data Availability

The data and supportive information are available within the article.

## LIST OF ABBREVIATIONS

SR: Stiffness Ratio
STE: Sound Touch Elastography
SWE: Shear Wave Elastography
NCCN: National Comprehensive Cancer Network
CT: Computer Tomography
MRI: Magnetic Resonance Imaging
TRUS: Transrectal Ultrasound
SE: Strain Elastography
ROI: Region of Interest
M-STB: Motion Stability Index
SD: Standard Deviation
IQR: Interquartile Range
AUC: Area Under the Curve
ROC: Receiver Operating Characteristic

## STANDARDS OF REPORTING

STROBE guidelines were followed.
